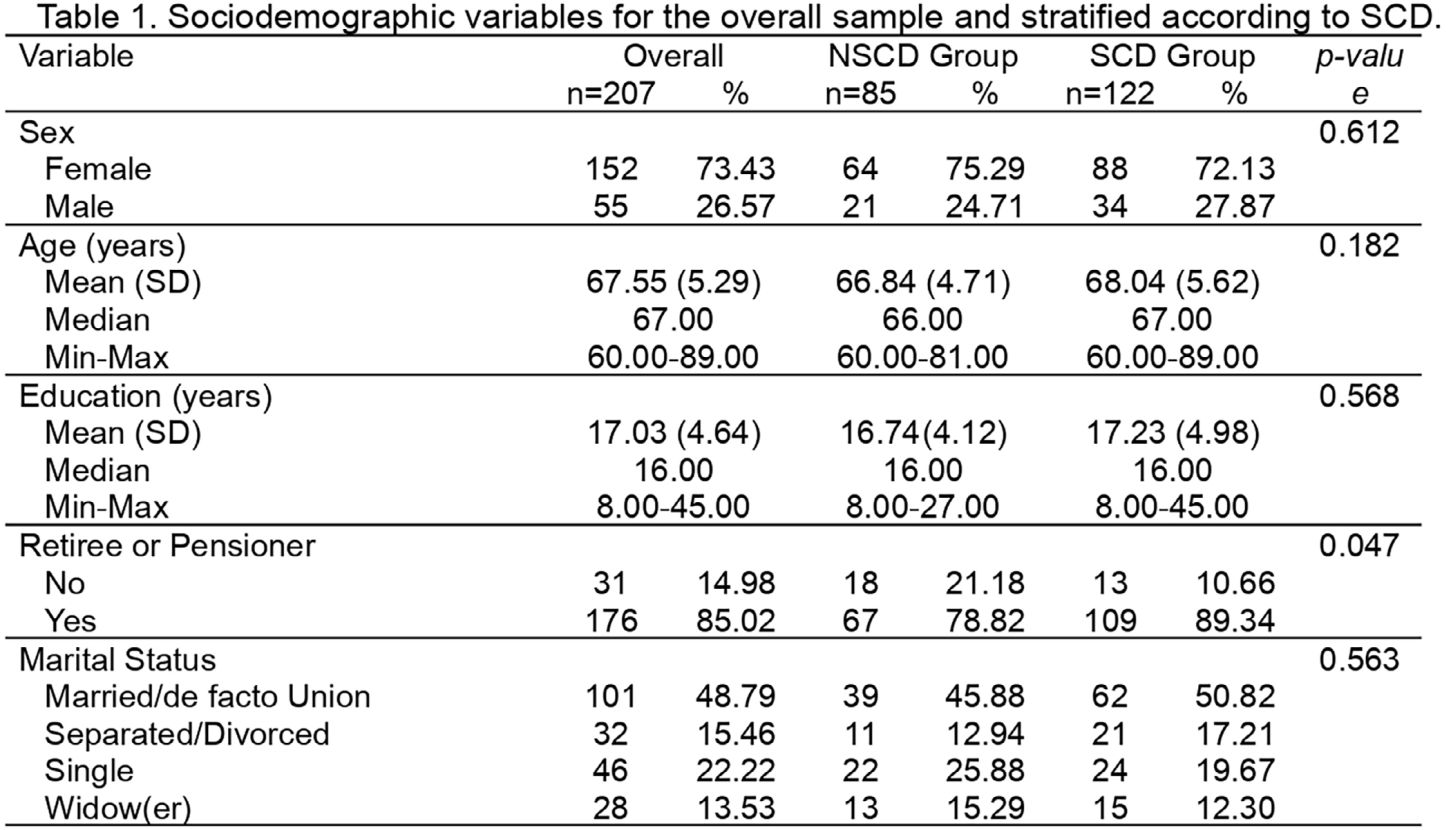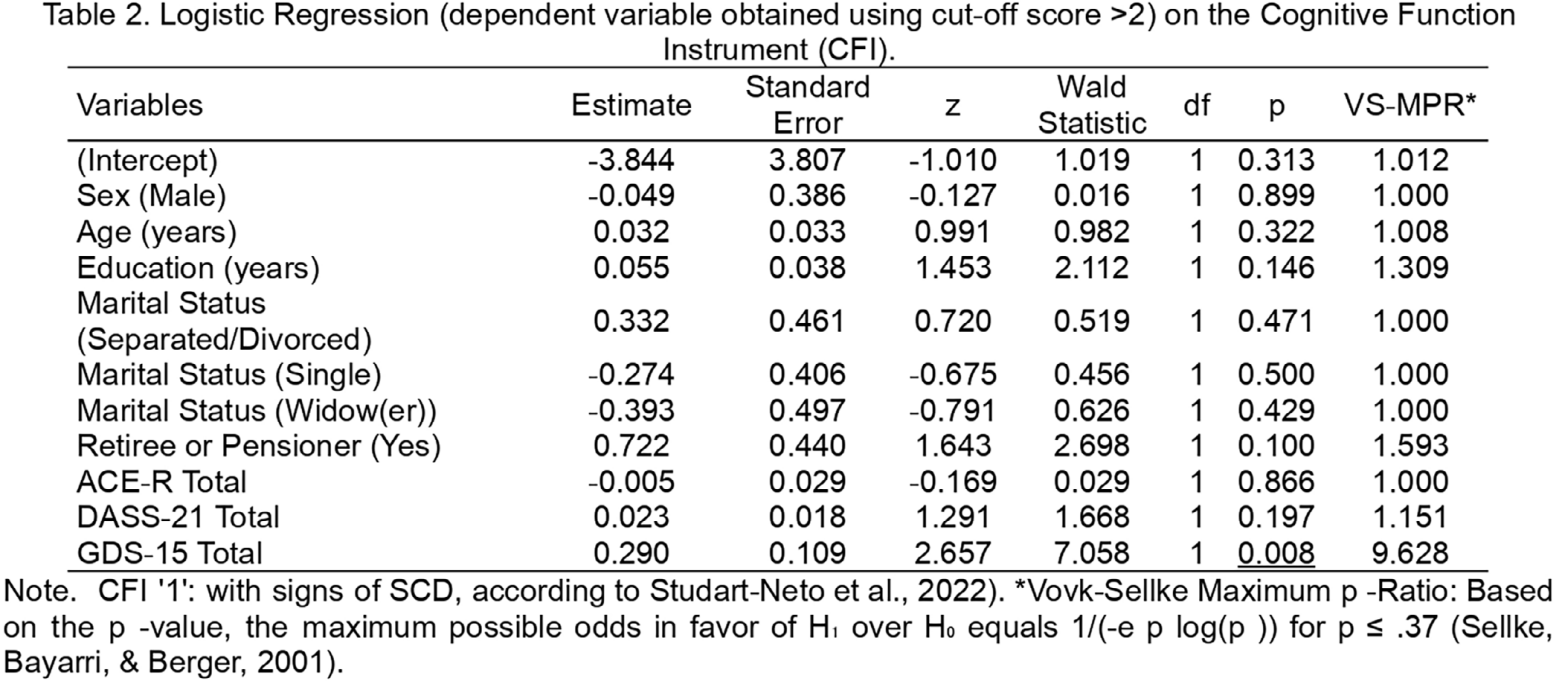# Subjective cognitive decline, depression, and anxiety in community‐dwelling older adults: a cross‐sectional study

**DOI:** 10.1002/alz70857_101529

**Published:** 2025-12-25

**Authors:** Gabriela dos Santos, Thais Bento Lima‐Silva, Tiago Nascimento Ordonez, Karen Jardim, Ana Paula Bagli Moreira, Laydiane Alves Costa, Diana Dos Santos Bacelar, Maria Antônia Antunes Fernandes, Sabrina Aparecida Da Silva, Henrique Salmazo da Silva, Beatriz Aparecida Ozello Gutierrez, Adalberto Studart‐Neto, Monica Sanches Yassuda, Sonia Maria Dozzi Brucki

**Affiliations:** ^1^ Universidade de São Paulo, São Paulo, São Paulo, Brazil; ^2^ Medical School of University of São Paulo, São Paulo, São Paulo, Brazil; ^3^ Gerontology of the School of Arts, Science and Humanities of the University of São Paulo, São Paulo, São Paulo, Brazil; ^4^ Universidade Católica de Brasília, Brasília, Brasília, Brazil

## Abstract

**Background:**

The rise in cases of cognitive decline and dementia in developing countries is concerning. Subjective Cognitive Decline (SCD), characterized by self‐perceived cognitive decline without impairment on neuropsychological tests, may be a preclinical stage of mild cognitive impairment. However, SCD prevalence in developing countries remains little investigated and standardized methods of assessment are lacking. Objective: To investigate the relationship among SCD, cognitive performance and mood variables in older adults.

**Method:**

207 older adult members of community centers and retiree associations were assessed. Participants completed the following assessment tests: the Cognitive Function Instrument (CFI) for measuring SCD, the Addenbrooke´s Cognitive Examination‐ Revised (ACE‐R) for assessing cognitive performance, and both the Geriatric Depression Scale ‐ 15 (GDS15) and the Depression, Anxiety and Stress Scale (DASS‐21) for assessing depressive and anxious symptoms.

**Result:**

Participants were classified as No‐SCD (CFI: M=1.34, DP = 0.68) or SCD (CFI: M=5.15, SD=1.98). Both groups were similar for age (No‐SCD: M=66.84, SD=4.71; SCD: M=68.04, 5.62), education (No‐SCD: M=16.74, SD=4.12; SCD: M=17.23. SD=4.98) and cognitive performance on the ACE‐R (No‐SCD: M=90.86, SD=4.98; SCD: M=90.08, SD=6.34). Differences were observed for retirement status (No‐SCD: *n* = 67; SCD: *n* = 109; *p* = 0.047) visuospatial subscale of the ACE‐R (No‐SCD: M=14.11, SD:1.74; SCD: M=14.70, SD:1.61; *p* = 0.009), DASS‐21 (No‐SCD: M=12.99, SD=10.29; SCD: M=18.44, SD=11.63; *p* <0.001) and on the GDS15 (No‐SCD: M=1.76, SD=1.73; SCD: M=2.84, SD=1.84; *p* <0.001). Also, multiple logistic regression analysis showed that the GDS15 score was statistically significant (*p* = 0.008), with an estimated positive coefficient of 0.290, indicating that higher scores on this variable were associated with greater odds of belonging to the SCD category.

**Conclusion:**

The results suggest depressive and anxious symptoms in older adults might be factors associated with SCD, highlighting the potential importance of mental‐health interventions in this group.